# Protective Effects of Velvet Antler Polypeptides on Cyclophosphamide-Induced Myelosuppression in Mouse and Bone Marrow Mesenchymal Stem Cells

**DOI:** 10.3390/nu17213428

**Published:** 2025-10-31

**Authors:** Fusheng Gao, Yusu Wang, Jinze Liu, Yichen Xie, Ying Geng, Zhongmei He, Jianan Geng, Jianming Li, Weijia Chen, Rui Du

**Affiliations:** 1 College of Chinese Medicinal Materials, Jilin Agricultural University, Changchun 130118, China; 2 Jilin Provincial Engineering Research Center for Efficient Breeding and Product Development of Sika Deer, Changchun 130118, China; 3 Key Laboratory of Animal Production and Product Quality and Security, Ministry of Education, Ministry of National Education, Changchun 130118, China

**Keywords:** myelosuppression, hematopoiesis, bone marrow microenvironment, cyclophosphamide

## Abstract

**Background:** Myelosuppression is one of the most common chemotherapy side effects, seriously threatening the quality of life of cancer patients. Studies have shown that velvet antler polypeptides (VAPs) could enhance immunity and anti-aging and also have a hematopoietic-promoting effect. However, there are relatively few studies on the treatment of myelosuppression with VAPs, and the therapeutic mechanism remains unclear. **Methods:** This study employed both in vitro and in vivo models to explore the mechanism of VAPs against myelosuppression. In this study, the cyclophosphamide (CTX)-induced bone marrow mesenchymal stem cell (BMSC) injury model was used to evaluate the effects of VAPs on cell viability, apoptosis, reactive oxygen species activity, and protein expression. Furthermore, a CTX-induced myelosuppression mouse model was employed to evaluate peripheral blood counts, organ indices, femoral tissue histopathology, immunohistochemical expression of CD34, VEGF, and Notch1, and key proteins in the Notch1/PI3K/AKT pathway in vivo. **Results:** Our results showed that VAPs protected BMSCs from CTX-induced apoptosis, inhibited ROS production, and promoted the secretion of VEGF, TPO, and VCAM-1, thereby improving the bone marrow microenvironment. Furthermore, the results showed that VAPs improved the peripheral blood counts and bone marrow nucleated cell (BMNC) count in CTX-induced myelosuppression mice and ameliorated pathological injury of the spleen, thymus, and liver. VAPs inhibited the apoptosis of bone marrow cells, manifested by regulating the expression levels of proteins like PI3K/p-PI3K, AKT/p-AKT, Bcl-2, Bax, and Caspase-3. Simultaneously, it upregulated the expression of Notch1 and Hes1 proteins. The application of the PI3K inhibitor LY294002 and the Notch1 inhibitor DAPT demonstrated that the ameliorative effect of VAPs on myelosuppression was dependent on the activation of both the Notch1 and PI3K/AKT pathways. **Conclusions:** Our study indicates that VAPs may achieve treatment of myelosuppression by improving the hematopoietic microenvironment, inhibiting apoptosis of mouse bone marrow cells, and regulating the Notch1 and PI3K/AKT signaling pathways.

## 1. Introduction

Cancer persists as a critical public health challenge and a leading cause of mortality globally. The most recent data from the World Health Organization (2022) evidenced more than 20 million new incident cases and approximately 9.7 million deaths [1]. Clinical treatment methods for cancer mainly include chemotherapy, radiotherapy, surgery, etc., among which chemotherapy is the most widely used treatment method for cancer [2]. However, most chemotherapeutic drugs, such as CTX, cisplatin, paclitaxel, etc., can induce side effects, such as myelosuppression [3]. The clinical manifestations of myelosuppression include anemia, pancytopenia, bleeding, infection, etc. [4]. These side effects will affect the treatment process of cancer patients and can even be life-threatening in severe cases [5]. Although drugs for the treatment of myelosuppression, such as granulocyte colony-stimulating factor (G-CSF) and granulocyte–macrophage colony-stimulating factor (GM-CSF), are effective in anti-myelosuppression, their disadvantages, such as high price, inducing ostealgia, dysphoria, and potential stimulation of malignant cell growth, limit their clinical application [6]. Therefore, it is very important to find and study drugs that improve myelosuppression and promote hematopoietic function recovery.

**Figure 1:**
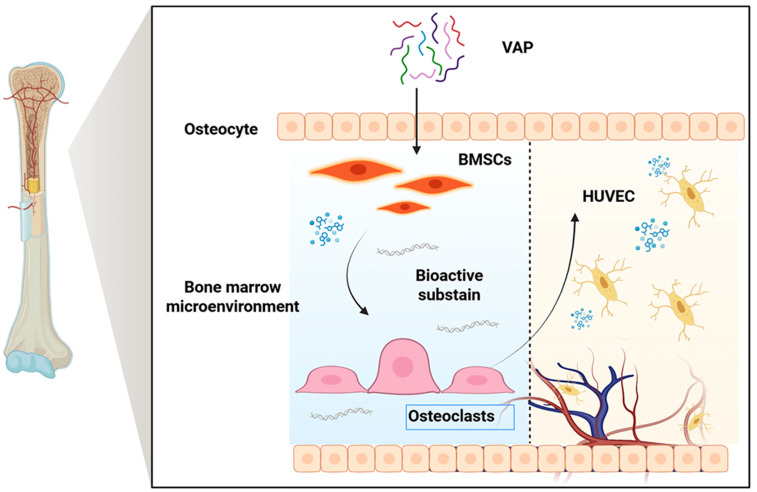
Schematic diagram of the proposed mechanism through which VAPs promote hematopoiesis.

Velvet antler is the only completely renewable mammalian organ, and it has become a research hotspot. It has been used in China, Japan, and South Korea for thousands of years [7]. VAP is one of the main components of velvet antler, which has the characteristics of small molecular weight, simple structure, easy uptake, and utilization by cells, so it is widely used in clinical practice [8]. In recent years, people have conducted a large number of pharmacological studies on VAPs and found that VAPs have biological activities in anti-osteoporosis [9], protection against cerebral ischemic injury [10], anti-aging [11], anti-depression [12], enhancing immunity [13], and other aspects. It is noteworthy that the bone marrow microenvironment plays a key role in the immune system and hematopoietic function. BMSC is an important part of the bone marrow microenvironment, which can regulate the proliferation, differentiation, and maturation of hematopoietic cells and is closely related to bone marrow hematopoietic function [14]. Previous studies have confirmed that VAPs can promote the proliferation and osteoblast differentiation of BMSCs and stimulate blood vessel generation [8,15], which provides a theoretical basis for VAPs in regulating the bone marrow microenvironment and promoting hematopoiesis (Figure 1).

This study aims to explore the mechanism of VAPs in alleviating myelosuppression by establishing a CTX-induced BMSC injury model in vitro and a mouse myelosuppression model in vivo. The therapeutic effects of VAPs were assessed through evaluations of apoptosis, ROS levels, complete blood count analysis, histopathological examination, and Western blotting. Our findings are expected to provide a solid experimental and theoretical foundation for developing VAPs as a novel therapeutic strategy for myelosuppression.

## 2. Materials and Methods

### 2.1. Reagents and Materials

Fresh velvet antler comes from sika deer, sourced from Shuangyang, Jilin (Jilin, China). Mouse vascular endothelial growth factor (VEGF) kits (Cat. No. SU-BN20737), mouse thrombopoietin (TPO) kits (Cat. No. SU-BN20763), and mouse vascular cell adhesion molecule 1 (VCAM-1) kits (Cat. No. SU-BN20741) were purchased from Huangshi Yanko Biotechnology Co., Ltd. (Huangshi, China). PI3K (Cat. No. WL02240), p-PI3K (Cat. No. WL03380), AKT (Cat. No. WL0003b), p-AKT (Cat. No. WLP001a), Bax (Cat. No. WL01637), Bcl-2 (Cat. No. WL01556), Hes1 (Cat. No. WL06387), and Notch1 (Cat. No. WL03097) antibodies were purchased from Shenyang Wanlei Biotechnology Co., Ltd. (Shenyang, China). BCA protein assay kits (Cat. No. R21250), LY29400 (Cat. No. S43088), and CTX (Cat. No. B24131) were purchased from Shanghai Yuanye Biotechnology Co., Ltd. (Shanghai, China). β-actin antibody (Cat. No. GB15003-100), CCK-8 (Cat. No. G4103-5ML), HRP conjugated Goat Anti-Rabbit IgG (Cat. No. GB23303), ROS detection kits (Cat. No. G1706-100T), and protein-free rapid blocking solution (Cat. No. G2052-500ML) were purchased from Wuhan Seville Biotechnology Co., Ltd. (Wuhan, China). N-[N-(3,5-Difluorophenacetyl)-L-alanyl]-S-phenyl-glycine t-butyl ester (DAPT) was purchased from Shanghai Haoyuan Biotechnology Co., Ltd. (Cat. No. HY-13027, Shanghai, China). Recombinant human granulocyte colony-stimulating factor (rhG-CSF) was purchased from Sino Biological Inc. (Cat. No. 10007-H01H, Beijing, China).

### 2.2. Experimental Animals

Sprague-Dawley male rats (3–4 weeks old) and 56 Kunming male mice (20 ± 2 g) were purchased from Changchun Yisi Laboratory Animal Technology Co., Ltd. (Changchun, China). All mice were housed under standard laboratory conditions with a room temperature of 20–25 °C, a relative humidity of 40–60%, and a 12 h light–dark cycle, with free access to food and water. The Laboratory Animal Welfare and Ethical Review Committee of Jilin Agricultural University reviewed this research project and determined that it met the ethical requirements for laboratory animals. The ethical review acceptance number was 20211011003 (Laboratory Animal License of the Laboratory Animal Center of Jilin Agricultural University: SYXK (ji) 2018–2023).

### 2.3. Preparation and Amino Acid Composition Analysis of VAPs

Fresh velvet antler was cut into blocks, freeze-dried, and then ground into powder. A measured amount of the velvet antler powder was weighed, and distilled water was added at a 1:15 ratio. Alkaline protease was added at 5000 U/g, and the pH was subsequently adjusted to 10 using a 1M sodium hydroxide solution. The mixture was enzymatically hydrolyzed in a 50 °C water bath for 2 h and then placed in a 100 °C water bath for 10 min to inactivate the enzyme. Finally, after centrifugation at 12,000× *g* for 20 min, the supernatant was collected as the VAPs’ extract. VAPs were purified using Sephadex G-25 gel filtration chromatography and then freeze-dried for further analysis.

### 2.4. Amino Acid Analysis

First, 6 mol/L of hydrochloric acid solution was added to the VAP samples and then hydrolyzed under nitrogen at 110 °C for 24 h. It was then diluted with distilled water, vacuum-dried, and dissolved in 0.02 mol/L of hydrochloric acid [16]. Subsequently, the amino acid composition was analyzed using an amino acid analyzer (L8900, Hitachi, Tokyo, Japan).

### 2.5. Cell Experiments

#### 2.5.1. Isolation and Culture of BMSCs

SD rats aged 3–4 weeks were selected as the source of primary rat bone marrow mesenchymal stem cells for in vitro cell experiments. The rats were anesthetized via intraperitoneal injection of pentobarbital sodium (50 mg/kg). Following complete anesthesia, euthanasia was performed through cervical dislocation. All femurs and tibias were removed on a sterile workbench, and alpha minimum essential medium (α-MEM, Gibco, Grand Island, NY, USA) containing 10% fetal bovine serum (FBS) supplemented with 10% FBS was aspirated with a sterile syringe and slowly flushed from top to bottom through the intramedullary space. The collected α-MEM supplemented with 10% FBS containing bone marrow fluid was centrifuged to discard the supernatant, transferred to cell culture flasks, and placed in a cell culture incubator for culture. After 24 h, nonadherent cells were removed by rinsing twice with phosphate-buffered saline (PBS). The adherent cells were maintained in α-MEM containing 10% FBS and 1% penicillin and streptomycin (PS) [17]. Bone marrow mesenchymal stem cells passaged to the 3rd–5th generation can be used for subsequent experimental research.

#### 2.5.2. Flow Cytometry

Bone marrow mesenchymal stem cells were washed with phosphate-buffered saline (PBS) and collected using trypsin. The cells were centrifuged twice at 1500 rpm. The cells were resuspended and incubated with a solution containing anti-CD11b, CD90, CD34, and CD45 antibodies for 30 min. After incubation with the antibodies, the cells were centrifuged at 1500 rpm for 5 min. The labeled cells were washed three times with PBS. Then, the labeled cells were resuspended in 200 μL of PBS and analyzed through flow cytometry.

#### 2.5.3. CCK-8 Assay for Proliferation Activity of BMSCs

BMSCs in the logarithmic growth phase were seeded into 96-well plates at a density of 5 × 10^4^ cells per well, with six replicate wells per group and a final volume of 100 μL per well. After 24 h of adhesion, the culture medium was replaced with α-MEM containing 1% FBS and supplemented with VAPs at concentrations of 0, 50, 100, 200, 400, 800, 1000, and 2000 μg/mL. After incubation in an incubator for 24 h, 10 μL of CCK-8 solution was added to each well, and absorbance was measured at 450 nm using a microplate reader. OD values were recorded, and cell viability was calculated. The maximum non-toxic dose to bone marrow mesenchymal stem cells was selected as the high dose for subsequent experiments, and half of this dose was used as the low dose.

#### 2.5.4. Cell Grouping and Treatment

An injury model of BMSCs was induced using CTX at a concentration of 15 mM for 2 h. BMSCs were randomly divided into seven groups: control group, model group (CTX (15 mM)), VAPs-L group (CTX + 200 μg/mL VAPs), VAPs-H group (CTX + 400 μg/mL VAPs), LY294002 group (CTX + 400 μg/mL VAPs + LY294002, a PI3K inhibitor), and positive control group (CTX + rhG-CSF). After successful model establishment, the corresponding drugs were added to each treatment group and incubated for 24 h to investigate the pharmacodynamic effects of VAPs.

#### 2.5.5. ELISA Detection of VEGF, TPO, and VCAM-1 Content in Bone Marrow Mesenchymal Stem Cells

After culturing the above groups of cells for 24 h, the supernatant was collected, centrifuged at 4 °C and 2000–3000 rpm for 20 min, and then aspirated. The ELISA kit instructions were followed to detect the content of VEGF, TPO, and VCAM-1 in the cell supernatant.

#### 2.5.6. Determination of Intracellular ROS Levels

After the cells were treated, the culture medium was removed, and the cells were washed once or twice with PBS buffer. Then, 1000 μL of DCFH-DA working solution was added to each well, and the plates were incubated in the dark at 37 °C in a CO_2_ incubator for 30 min. Following incubation, the DCFH-DA working solution was aspirated, and the cells were washed two to three times with PBS to thoroughly remove any excess probe. Finally, the cells were covered with PBS and observed under a fluorescence microscope.

#### 2.5.7. Hoechst33342/PI Staining Method for Apoptosis Detection

After drug treatment, 5 μL of Hoechst33342, 5 μL of PI, and 1 mL of cell staining buffer were added to each well, followed by 20 min of incubation. Subsequently, the cells were washed with PBS and observed under a fluorescence microscope.

### 2.6. Animal Experiments

#### 2.6.1. Animal Grouping and Treatment

All mice were acclimated and fed for 7 days prior to administration under normal light and humidity conditions, with free access to water and food. A total of 56 mice were randomly divided into 7 groups (*n* = 8). Except for the control group, mice in the other groups received an intraperitoneal injection of a certain dose of CTX (100 mg/kg) for 3 days, while the control group received an equal volume of normal saline. Then, 24 h after CTX treatment, the positive drug (rhG-CSF) group received an intraperitoneal injection (11.25 μg/kg), the low-dose treatment (VAPs-L) group and the high-dose (VAPs-H) treatment group received intragastric administration (100 mg/kg and 200 mg/kg, respectively), the LY294002 group received VAPs (200 mg/kg) and LY294002 (10mg/kg), and the DAPT group received VAPs (200 mg/kg) and DAPT (10mg/kg). The control group and the model group were given an equal volume of normal saline once daily for 14 consecutive days.

#### 2.6.2. Peripheral Blood Analysis

Following treatment, all mice were euthanized through intraperitoneal injection of 1% pentobarbital (65 μL/10 g). Blood samples were collected from the retroorbital venous plexus of all mice and injected into both ethylenediaminetetraacetic acid (EDTA)-anticoagulated tubes and standard blood collection tubes. Following collection, the standard blood collection tubes were immediately centrifuged (3500 rpm, 10 min, 4 °C). The resulting serum was stored at −80 °C for subsequent analysis. Blood samples in EDTA tubes were immediately used for complete blood cell counts.

#### 2.6.3. Organ Index

After the mice were sacrificed, the spleen, thymus, and liver were isolated, rinsed with saline to remove blood traces, and gently blotted dry on filter paper. The organs were then weighed to record their mass. The thymus index (TI), spleen index (SI), and liver index (LI) were then calculated.

Organ index (%) = [Organ weight (mg)/Body weight (g)] × 100%.

#### 2.6.4. Hematoxylin and Eosin Staining (HE)

The femur was fixed with 4% paraformaldehyde for 24 h, followed by decalcification using 10% EDTA decalcifying solution. After decalcification, the samples were embedded and sectioned. After deparaffinization with a xylene gradient and dehydration with an ethanol gradient, HE staining was performed. The pathological condition of the femur tissue was observed under a Leica DM1000 light microscope (Leica Microsystems, Wetzlar, Germany).

Liver and spleen tissues were collected from mice and fixed with 4% paraformaldehyde, followed by embedding and sectioning. After deparaffinization with a xylene gradient and dehydration with an ethanol gradient, HE staining was performed. The morphological characteristics of liver and spleen tissues in each group were observed under a microscope (Leica Microsystems, Wetzlar, Germany).

#### 2.6.5. Detection of VEGF, TPO, and VCAM-1 Levels in Serum

The levels of VEGF, TPO, and VCAM-1 in the serum were measured according to the instructions provided with the ELISA detection kit.

#### 2.6.6. BMNC Count

After the femurs were isolated, the epiphyses at both ends were removed. The bone marrow was flushed from the medullary cavity with PBS into a centrifuge tube. Subsequently, erythrocyte lysis buffer was added to remove red blood cells. After centrifugation, the supernatant was discarded. The resulting cell pellet was resuspended in 1 mL of PBS, and the nucleated cells were counted using an optical microscope (Leica Microsystems, Wetzlar, Germany).

#### 2.6.7. Immunohistochemical Analysis

Femur tissue was fixed in 4% paraformaldehyde, dehydrated with ethanol, embedded in paraffin wax, and sectioned. After antigen retrieval and blocking with bovine serum albumin (BSA), CD34 Rabbit pAb (1:500), VEGF (SP07-01, 1:400), and Notch1 Rabbit pAb (1:200) antibodies were added and incubated overnight, followed by incubation with HRP-conjugated Goat Anti-Rabbit IgG (1:200) for 2 h. DAB chromogenic solution was added, and hematoxylin was used for counterstaining. The expression of CD34, VEGF, and Notch1 proteins in femur tissue was observed under a light microscope. Three high-expression areas were selected for imaging and analyzed using Image J Pro-Plus 6.0 software.

### 2.7. Western Blot

BMSCs and bone marrow cells were collected from mouse femurs in each group. Total protein was extracted using RIPA lysis buffer, and the protein concentration of the samples was determined using the BCA assay. Approximately 10 μg of protein per lane was loaded and separated through electrophoresis on a 10% SDS-PAGE gel. After electrophoresis, the separated target proteins were transferred onto a polyvinylidene fluoride (PVDF) membrane using the wet transfer method. The membrane was then blocked with a protein-free rapid blocking solution at room temperature for 30 min. Subsequently, it was incubated overnight at 4 °C with the following primary antibodies: PI3K (1:1500), p-PI3K (1:800), AKT (1:800), p-AKT (1:800), Bax (1:1500), Bcl-2 (1:1000), and β-actin (1:4000). After incubation, the membrane was washed three times with TBST for 10 min each. An HRP-labeled secondary antibody (1:8000) was applied and incubated for 2 h at room temperature. Finally, the blot was exposed using the Tanon ECL Chemiluminescent Substrate (Cat. No. 180-5001), and images were captured. The grayscale values of the bands were analyzed using ImageJ Pro-Plus 6.0 software, and the ratio of the target protein to the internal reference β-actin was used as its relative content. It is worth noting that band intensity detected by phosphorylation-specific antibodies serves as an indirect measure of kinase (re)activation.

### 2.8. Statistical Analysis

All data are expressed as mean ± SD. Differences between groups were analyzed using one-way analysis of variance (ANOVA) followed by a *t*-test. Data were statistically analyzed using GraphPad Prism 9.5.1. *p* < 0.05 was considered statistically significant.

## 3. Results

### 3.1. Amino Acid Composition of VAPs

**Figure 2:**
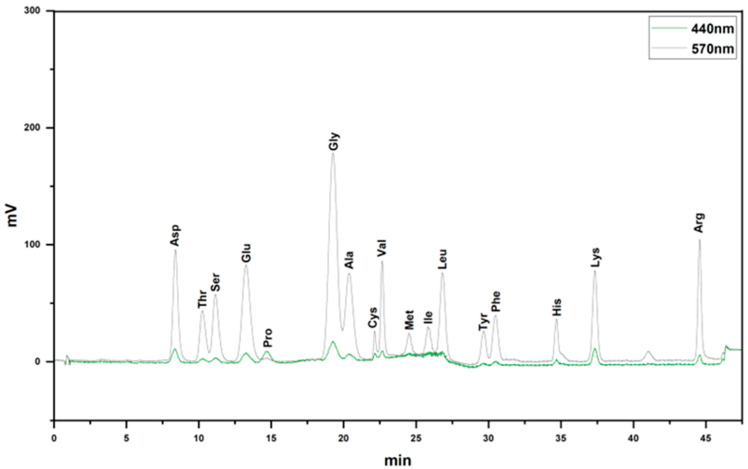
Amino acid composition of VAPs.

Through amino acid analysis, it was discovered that VAPs mainly contained Asp, Thr, Ser, Glu, Pro, Gly, Ala, Cys, Val, Met, Ile, Leu, Tyr, Phe, His, Lys, and Arg (Figure 2 and Table 1).

### 3.2. Characterization of BMSCs

**Figure 3:**
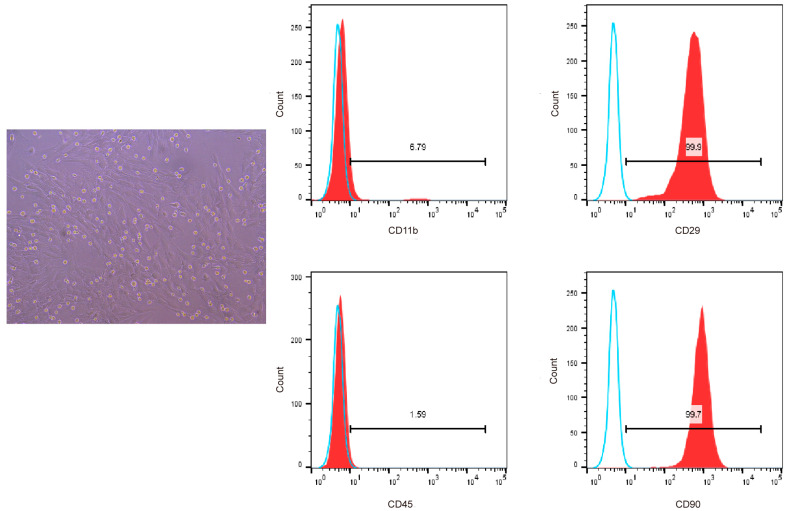
Flow cytometric analysis of CD11b, CD45, CD29, and CD90 surface marker expression on BMSCs. Blue solid lines represent the negative control while red solid lines represent the expression of surface markers.

We used flow cytometry to detect the surface markers of BMSCs to confirm that the cells isolated and cultured from the bone marrow of SD rats were bone marrow mesenchymal stem cells. The results showed that the cells highly expressed CD29 and CD90, which are specific surface markers of stem cells [18], and the positive cell rate was greater than 94%, with low expression of CD11b and CD45. Therefore, we successfully isolated bone marrow mesenchymal stem cells from SD rats (Figure 3).

### 3.3. The Effects of VAPs on VEGF, TPO, and VCAM-1 in CTX-Induced BMSCs

**Figure 4:**
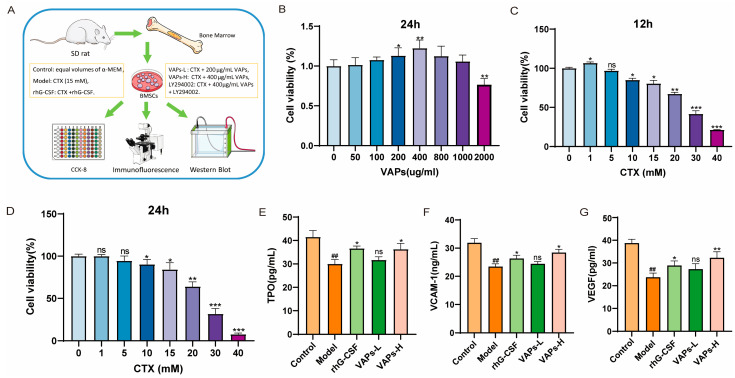
VAPs enhance the secretion of hematopoietic factors in CTX-induced BMSCs. (**A**) Cell experiment procedure diagram. (**B**) The effect of VAPs on cell viability. (**C**,**D**) Effect of CTX on cell viability at different time points (12/24 h). (**E**–**G**) The effect of VAPs on the secretion of VCAM-1, VEGF, and TPO in CTX-induced BMSCs. ^##^ *p* < 0.01 vs. the control group; * *p* < 0.05, ** *p* < 0.01, *** *p* < 0.001 vs. the model group; ns, not significant (*p* > 0.05).

Figure 4A shows the cell experiment flowchart and grouping status. After treatment with VAPs for 24 h, cell viability showed a trend of initial increase followed by a decrease, with the highest viability observed at 400 μg/mL VAPs, as shown in Figure 4B. When BMSCs were treated with different concentrations of CTX for 12 or 24 h, cell viability initially increased and then decreased, showing dose dependence. The concentration of CTX that resulted in approximately 70% cell viability was selected as the modeling concentration (Figure 4C,D). Therefore, concentrations of 200 μg/mL and 400 μg/mL VAPs and a modeling concentration of 15 mM CTX were used for the pharmacodynamic experiments.

BMSCs play a crucial role in supporting and regulating hematopoiesis within the bone marrow hematopoietic microenvironment. We measured the levels of hematopoiesis-related factors in the culture supernatant of BMSCs from each group. VEGF is one of the most important regulatory factors in blood vessel development and angiogenesis [19], and it enhances oxygen supply to hematopoietic cells. TPO could induce the maturation and differentiation of megakaryocytes, promoting the production of megakaryocytes and thrombocytes [20]. VCAM-1 can enhance the adhesion and homing of hematopoietic stem cells within the bone marrow microenvironment. These hematopoietic factors regulate the proliferation and differentiation of hematopoietic stem cells and maintain the homeostasis of the hematopoietic microenvironment [21]. As shown in Figure 4E,F, the levels of VEGF, TPO, and VCAM-1 in the model group were significantly decreased compared with the control group. Both VAPs and rhG-CSF treatment reversed this decrease. Notably, the restorative effect of VAPs exhibited a dose-dependent pattern. The VAPs-H group demonstrated effects comparable to the positive drug, while the VAPs-L group showed an upward trend without statistically significant difference.

### 3.4. Effects of VAPs on CTX-Induced Apoptosis and ROS Levels in BMSCs

**Figure 5:**
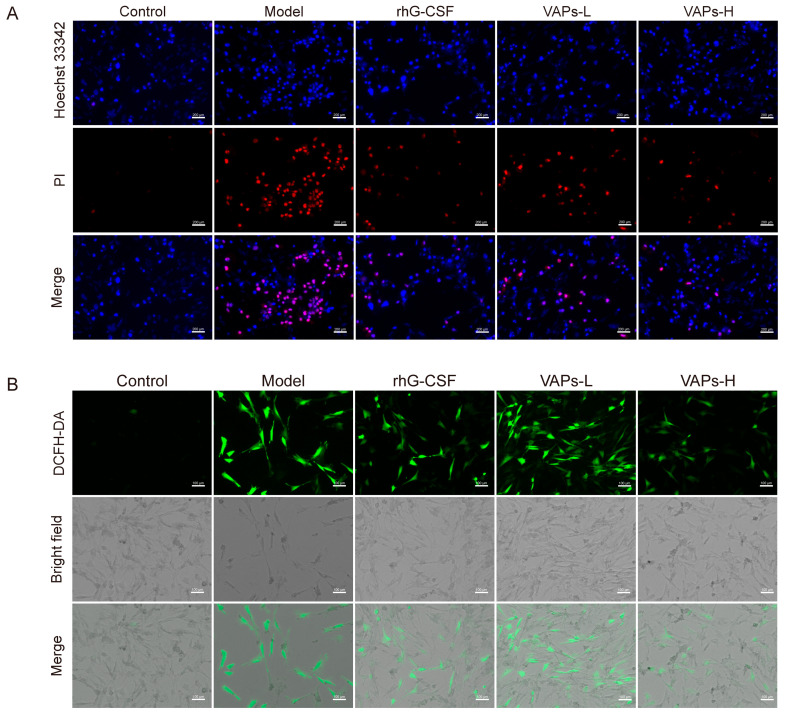
Effect of VAPs on ROS levels and intracellular apoptosis in BMSCs cells. (**A**) Hoechst/PI staining in BMSCs. (**B**) ROS staining in BMSCs.

We detected apoptosis through Hoechst 33342/PI staining. As shown in Figure 5A, a strong PI red fluorescence signal was observed in the model group compared to the control group, indicating an increased proportion of apoptotic BMSCs. In contrast, treatment with either VAPs or rhG-CSF significantly attenuated the PI fluorescence intensity and substantially reduced the area of red fluorescence in the visual field.

ROS are maintained at a low level through the fine-tuning of redox homeostasis to support normal signaling transduction. However, oxidative DNA damage can be induced by the aberrant accumulation of ROS, leading to cellular senescence and apoptosis [22], thereby inhibiting hematopoietic function. In the present study, a significant increase in intracellular ROS levels was observed in the model group, as illustrated in Figure 5B. This elevation was effectively reversed by treatment with either VAPs or rhG-CSF. These results suggest that VAPs exhibit anti-apoptotic and antioxidant effects, alleviating the injury induced by CTX on BMSCs.

### 3.5. Regulation of the PI3K/AKT Signaling Pathway Within BMSCs by VAPs

**Figure 6:**
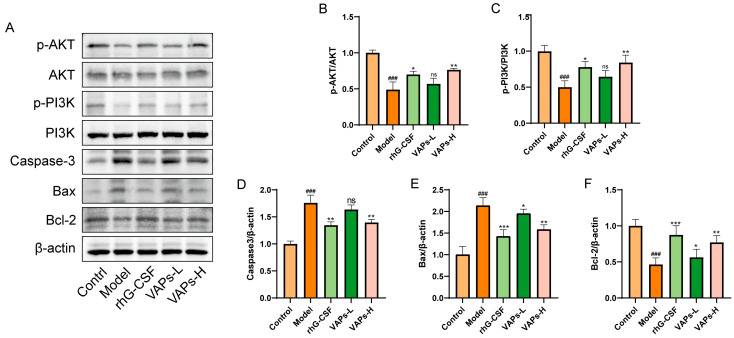
Effects of VAPs on the expression of AKT/PI3K, Caspase-3, Bax, and Bcl-2 in bone marrow tissue. (**A**) Western blot images. (**B**–**F**) Quantitative analysis of the p-AKT/AKT and p-PI3K/PI3K ratios, as well as the protein expression levels of Caspase-3, Bax, and Bcl-2. The phosphatidylinositol-3-kinase/protein kinase B (re)activation is nominal, although activity has been quantified via band intensity analysis. ^###^ *p* < 0.001 vs. the control group; * *p* < 0.05, ** *p* < 0.01, *** *p* < 0.001 vs. the model group; ns, not significant (*p* > 0.05).

The expression levels of relevant proteins in BMSCs were determined through Western blotting. As shown in Figure 6, the model group exhibited significantly downregulated ratios of p-PI3K/PI3K and p-AKT/AKT, as well as decreased Bcl-2 protein expression compared with the control group, whereas the levels of Caspase-3 and Bax were significantly upregulated. Following intervention with VAPs or rhG-CSF, these changes were effectively reversed. These results indicate that VAPs may exert their anti-apoptotic effects by activating the PI3K/AKT signaling pathway and regulating downstream apoptosis-related proteins.

### 3.6. The PI3K Inhibitor LY294002 Verified the Effect of VAPs on the Proteins of the PI3K/AKT Pathway in BMSCs

**Figure 7:**
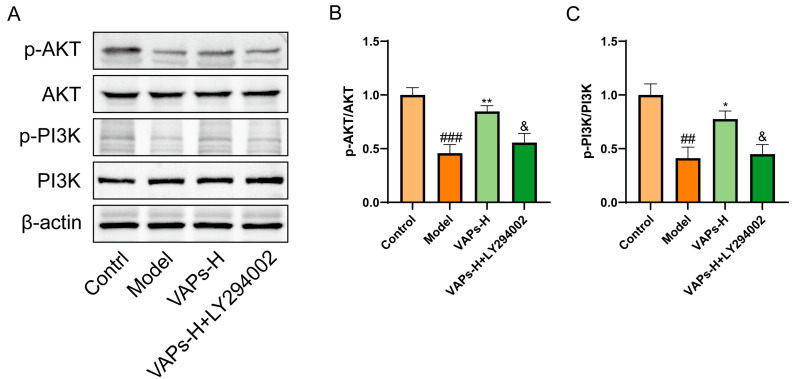
Pharmacological inhibition of PI3K by LY294002 abolishes the protective effects of VAPs in BMSCs. (**A**) Western blot images. (**B**,**C**) Quantitative analysis of the p-AKT/AKT and p-PI3K/PI3K ratios, as well as the protein expression levels of Caspase-3, Bax, and Bcl-2. Phosphatidylinositol-3-kinase/protein kinase B (re)activation is nominal, although activity has been quantified via band intensity analysis. ^##^ *p* < 0.01, ^###^ *p* < 0.001 vs. the control group; * *p* < 0.05, ** *p* < 0.01 vs. the model group; ^&^ *p* < 0.05 vs. the VAPs-H group.

Western blot results showed that LY294002 treatment reversed the upregulation of p-PI3K/PI3K and p-AKT/AKT induced by VAPs (Figure 7B,C). These findings suggest that VAPs may mitigate CTX-induced cellular damage in BMSCs by modulating the PI3K/AKT pathway.

### 3.7. General Condition of Mice

**Figure 8:**
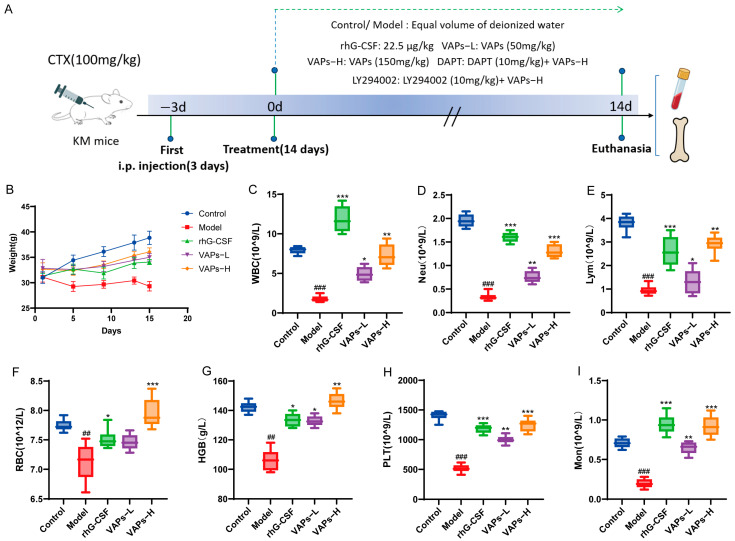
The experimental procedure and effects of VAPs on mouse body weight and peripheral complete blood count. (**A**) Diagrammatic illustration of the experimental procedure. (**B**) Changes in mouse body weight during the study. (**C**) White blood cell (WBC). (**D**) Neutrophil (Neu). (**E**) Lymphocyte (Lym). (**F**) Monocyte (Mon). (**G**) Red blood cell (RBC). (**H**) Hemoglobin (Hb). (**I**) Platelet (PLT). Compared with ctrl group ^##^ *p* < 0.01, ^###^ *p* < 0.001. Compared with model group * *p* < 0.05, ** *p* < 0.01, *** *p* < 0.001.

The overall experimental timeline and group allocation are summarized in Figure 8A. As shown in Figure 8B, control mice exhibited normal behavioral and physiological characteristics, including alert mental state, free movement, glossy fur, responsive reactions, normal feeding, and stable weight gain. In contrast, model group mice exhibited lethargy, matted fur, unsteady gait, significant hair loss, and delayed weight gain. Compared with the model group, mice in the rhG-CSF group and the VAPs group showed varying degrees of improvement in these conditions.

### 3.8. Peripheral Blood Cells

Peripheral blood cells can reflect hematopoietic function, and the occurrence of bone marrow hematopoietic dysfunction can be evaluated through peripheral blood tests. As shown in Figure 8C–G, the model group showed significant decreases in WBC, RBC, HGB, PLT, Neu, Lym, and Mon compared with the normal group, indicating that bone marrow hematopoietic function was markedly impaired. Treatment with VAPs produced a broad improvement in all hematopoietic lineages in myelosuppression mice. It is noteworthy that rhG-CSF exhibited a more pronounced effect on WBC, Neu, Lym, and Mon, which is related to rhG-CSF mainly promoting the proliferation and differentiation of bone marrow granulocytic hematopoietic progenitor cells. These results indicate that VAPs can effectively improve multilineage hematopoietic cells in myelosuppression mice.

### 3.9. Bone Marrow Nucleated Cell Count and Organ Index Analysis

**Figure 9:**
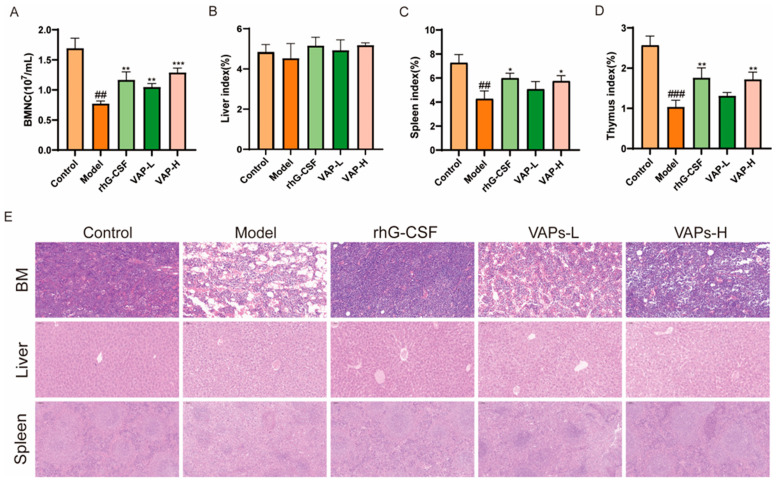
Effects of VAPs on bone marrow nucleated cell count, organ indices, and histopathological staining. (**A**) Bone marrow nucleated cell count (BMNC). (**B**–**D**) The organ indices of the liver, spleen, and thymus. (**E**) Hematoxylin–eosin staining results of mouse femoral bone marrow, liver, and spleen. Compared with ctrl group ^##^ *p* < 0.01, ^###^ *p* < 0.001. Compared with model group * *p* < 0.05, ** *p* < 0.01, *** p < 0.001.

As shown in Figure 9A, the number of BMNCs in the model group mice was significantly reduced compared with the control group. Compared with the model group, the number of BMNCs in each drug administration group was significantly restored. As important immune organs, the thymus and the spleen are prone to atrophy under the action of chemotherapy drugs, leading to damage to the immune system. Compared with the model group, the thymic and splenic indices were significantly increased following treatment with VAPs or rhG-CSF, indicating that VAPs can ameliorate CTX-induced atrophy of immune organs and exert an immune protective effect. However, no statistically significant differences were observed in the liver indices among the groups (Figure 9B–D).

### 3.10. VAPs’ Effects on the Histopathological Morphology of the Femur, Spleen, and Liver Tissues in Myelosuppression Mice

The results of HE showed that the model group exhibited characteristic tissue injuries, including a reduced number of disorganized cells in the femoral bone marrow cavity, disrupted liver lobule structure with chaotic hepatic cord arrangement, and blurred boundaries between the red and white pulp, along with a weakened lymphoid sheath in the spleen compared to the control group. These histopathological damages were ameliorated through treatment with either VAPs or rhG-CSF (Figure 9E).

### 3.11. Effects of VAPs on Hematopoietic-Related Cytokines in Myelosuppression Mice

**Figure 10:**
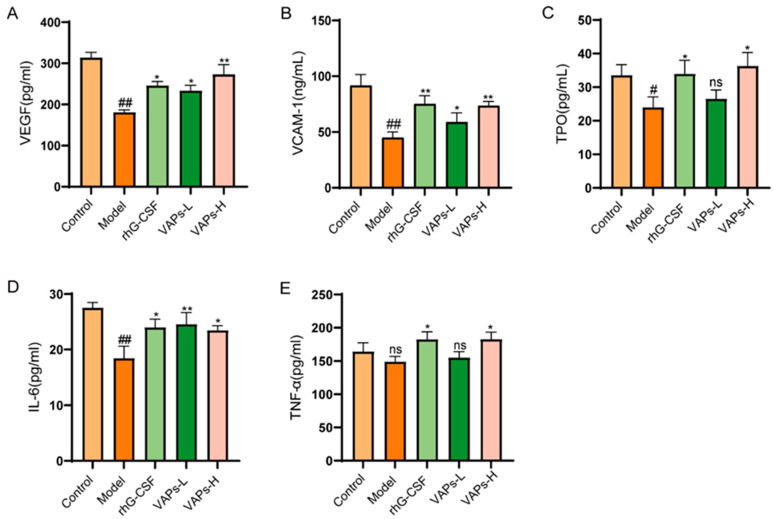
VAPs enhance hematopoiesis by upregulating serum levels of key hematopoietic cytokines in myelosuppression mice. (**A**) VEGF. (**B**) VCAM-1. (**C**) TPO. (**D**) IL-6. (**E**) TNF-α. Compared with ctrl group ^#^ *p* < 0.05, ^##^ *p* < 0.01; compared with model group * *p* < 0.05, ** *p* < 0.01; ns, not significant (*p* > 0.05).

CTX severely impairs hematopoietic function in mice, leading to reduced levels of hematopoietic-related cytokines. VEGF, VCAM-1, TPO, IL-6, and TNF-α, as key hematopoietic regulatory factors in the bone marrow microenvironment, all exhibited a decreasing trend in model group mice. VAPs could enhance the expression of the aforementioned cytokines (Figure 10), indicating that they may promote hematopoietic recovery by regulating key signaling molecules in the hematopoietic microenvironment.

### 3.12. Protein Expression of CD34, VEGF, and Notch1 in Bone Marrow Tissue

**Figure 11:**
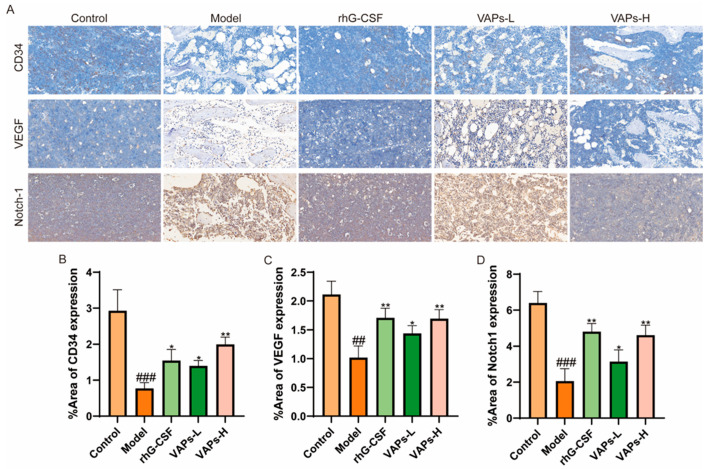
Expression of CD34, VEGF, and Notch-1 in bone marrow tissues, detected through immunohistochemistry. (**A**) Immunohistochemical staining of CD34, VEGF, and Notch1. (**B**–**D**) Quantitative analysis of the IHC results shown in panel A. ^##^ *p* < 0.01, ^###^ *p* < 0.001 vs. the control group; * *p* < 0.05, ** *p* < 0.01 vs. the model group.

As shown in Figure 11**,** immunohistochemical staining showed that the expression levels of CD34, VEGF, and Notch-1 proteins in the bone marrow tissue of the model group mice were significantly reduced compared with the normal group. The positive drug group and all dosage groups of VAPs upregulated the expression of these proteins to varying degrees. These findings suggest that VAPs may promote bone marrow microvascular formation in myelosuppression mice, regulate the Notch1 signaling pathway, improve the bone marrow microenvironment, and facilitate hematopoietic recovery.

### 3.13. VAPs’ Effects on the PI3K/AKT Pathway and Notch1 in Myelosuppression Mice

**Figure 12:**
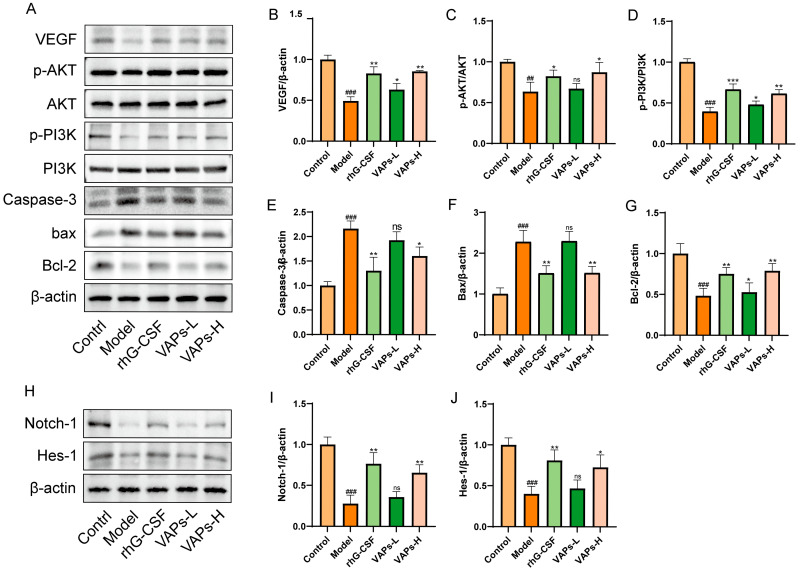
Effects of VAPs on the expression of PI3K/AKT, Caspase-3, Bax, Bcl-2, Notch-1, and Hes-1 in bone marrow tissue. (**A**,**H**) Western blot images. (**B**–**G**) Quantitative analysis of the p-AKT/AKT and p-PI3K/PI3K ratios, as well as the protein expression levels of Caspase-3, Bax, and Bcl-2. Phosphatidylinositol-3-kinase/protein kinase B (re)activation is nominal, although activity has been quantified via band intensity analysis. (**I**,**J**) The protein expression levels of Notch-1 and Hes-1. ^##^ *p* < 0.01, ^###^ *p* < 0.001 vs. the control group; * *p* < 0.05, ** *p* < 0.01, *** *p* < 0.001 vs. the model group; ns, not significant (*p* > 0.05).

As shown in Figure 12A–G, the protein expression levels of p-PI3K/PI3K, p-AKT/AKT, Bcl-2, and VEGF were significantly decreased in the model group, whereas the levels of Caspase-3 and Bax were markedly increased compared with the control group. These aberrant expression trends were effectively reversed following treatment with VAPs. To further explore the multi-pathway regulatory mechanism of VAPs, we examined the expression of key proteins in the Notch1 signaling pathway. The results showed that both VAPs significantly upregulated the expression of Notch1 and its downstream effector Hes1 compared to the model group (Figure 12H–J). These findings suggest that the PI3K/AKT and Notch1 signaling pathways collectively mediate the ameliorative effects of VAPs on myelosuppression.

### 3.14. The PI3K Inhibitor LY294002 Verified the Effect of VAPs on the Proteins of the PI3K/AKT Pathway

**Figure 13:**
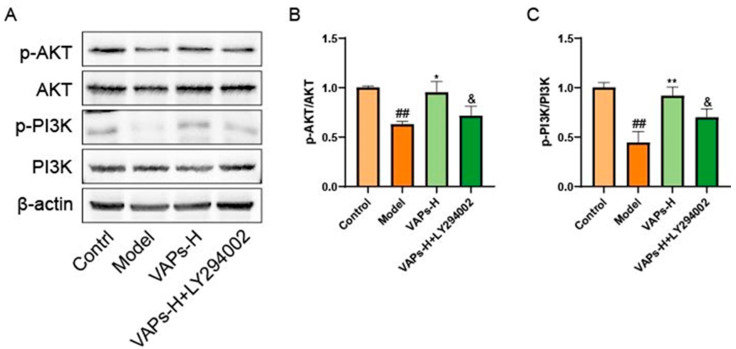
Pharmacological inhibition of PI3K by LY294002 abolishes the protective effects of VAPs in bone marrow tissue. (**A**) Western blot images. (**B**,**C**) Quantitative analysis of the p-AKT/AKT and p-PI3K/PI3K ratios, as well as the protein expression levels of Caspase-3, Bax, and Bcl-2. Phosphatidylinositol-3-kinase/protein kinase B (re)activation is nominal, although activity has been quantified via band intensity analysis. ^##^ *p* < 0.01 vs. the control group; * *p* < 0.05, ** *p* < 0.01 vs. the model group; ^&^ *p* < 0.05 vs. the VAPs-H group.

Consistent with our previous findings, the expression levels of p-PI3K/PI3K and p-AKT/AKT in bone marrow tissues were decreased in the model group. In contrast, VAP treatment significantly upregulated the ratios of p-PI3K/PI3K and p-AKT/AKT relative to the model group. However, administration of the PI3K inhibitor LY294002 abolished the therapeutic effects of VAPs (Figure 13).

### 3.15. The Notch1 Inhibitor DAPT Validated the Effects of VAPs on Notch1 and PI3K/AKT Pathway Proteins

**Figure 14:**
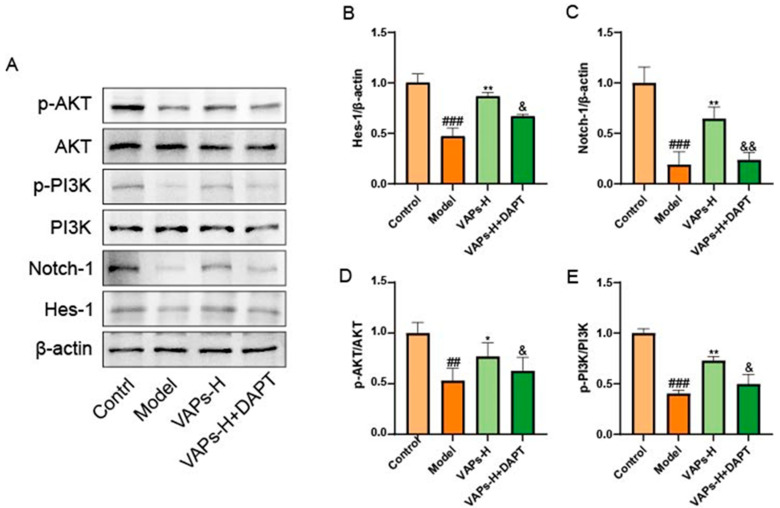
Pharmacological inhibition of Notch1 by DAPT abrogates the therapeutic effects of VAPs and their activation on the PI3K/Akt pathway. (**A**) Western blot images. (**B**–**E**) Quantitative analysis of the p-AKT/AKT and p-PI3K/PI3K ratios, as well as the protein expression levels of Hes-1 and notch-1. Phosphatidylinositol-3-kinase/protein kinase B (re)activation is nominal, although activity has been quantified via band intensity analysis. ^##^ *p* < 0.01, ^###^ *p* < 0.001 vs. the control group; * *p* < 0.05, ** *p* < 0.01 vs. the model group; ^&^ *p* < 0.05, ^&&^ *p* < 0.01 vs. the VAPs-H group.

Numerous studies have established that the Notch pathway can function as an upstream positive regulator of the PI3K/Akt signaling pathway. Inhibition of the Notch pathway by γ-secretase inhibitors (GSI) reduces the activation of the Notch and Akt/mTOR pathways [23,24,25]. Therefore, we hypothesized that the molecular mechanism of VAPs might positively regulate the PI3K/Akt pathway through modulation of the Notch1 pathway. To verify this hypothesis, we examined the expression of Notch1 and PI3K/Akt pathway proteins by adding the Notch1 inhibitor DAPT. As shown in Figure 14, inhibition of the Notch1 pathway led to a decrease in the expression of proteins related to the PI3K/Akt pathway and reversed the therapeutic effect of VAPs, indicating that VAPs may restore hematopoietic function by regulating the Notch1/PI3K/Akt pathway.

## 4. Discussion

The incidence of neoplasms has been increasing year by year. Chemotherapy, as a critical treatment for cancer, commonly leads to myelosuppression, which is one of the main reasons for dose reduction or discontinuation of chemotherapy drugs. This condition poses a serious threat to both the survival and quality of life of patients with neoplasms [26,27,28,29]. The drugs commonly used in clinical practice to treat myelosuppression are costly and often associated with multiple adverse effects [30]. Therefore, there is a pressing need to identify low-cost, safe, and effective medications to alleviate myelosuppression caused by chemotherapy and to restore bone marrow hematopoietic function. In this study, the therapeutic effects and potential mechanisms of VAPs on myelosuppression induced by CTX were investigated from the perspectives of bone marrow cell self-repair and regulation of the bone marrow microenvironment.

In this study, amino acid composition analysis comprised acid hydrolysis. This is the most commonly used method for total amino acid analysis of peptides. However, this method has inherent limitations. Trp is completely destroyed under acidic hydrolysis conditions and thus cannot be detected, and Asn and Gln lose their amide groups during acid hydrolysis, converting to Asp and Glu, respectively [31]. Therefore, the elevated Asp and Glu levels reported in our study actually incorporate portions derived from the conversion of Asn and Gln.

BMSCs are a key component of the bone marrow hematopoietic microenvironment. They support the differentiation and proliferation of hematopoietic stem cells [32], promote the formation of osteoblasts, osteoclasts, and blood vessels [33], and play a role in immune regulation [34]. Previous studies have shown that bone marrow mesenchymal stem cells can enhance the hematopoietic microenvironment through specific chemokines, facilitate the homing of transplanted hematopoietic stem cells, and help maintain hematopoietic homeostasis by secreting various cytokines [35,36]. CTX is a commonly used anticancer chemotherapy drug that can be administered alone or in combination with other chemotherapy agents [37,38]. Its metabolites, such as cyclophosphamide mustard and Acrolein, can cause DNA damage, induce apoptosis in bone marrow cells, and suppress hematopoietic function [39,40,41].

In this study, we used CTX to induce injury in BMSCs, thereby simulating the pathogenesis of in vitro myelosuppression. Our results demonstrated that CTX significantly decreased cell viability and triggered apoptosis and oxidative stress. In contrast, treatment with VAPs partially mitigated the CTX-induced damage to bone marrow mesenchymal stem cells, as reflected by enhanced cell proliferation, reduced apoptosis, restored antioxidant enzyme activity, and reversal of the suppression of VEGF, TPO, and VCAM-1 secretion caused by CTX.

The phosphatidylinositol-3-kinase/protein kinase B (PI3K/AKT) signaling pathway is a key intracellular signal transduction pathway with broad biological effects. It is involved in cellular processes like growth, proliferation, differentiation, apoptosis, and metabolism, and it plays an important role in maintaining hematopoietic homeostasis [42,43]. In the regulation of apoptosis, the PI3K heterodimer (composed of a p85 regulatory and a p110 catalytic subunit) functions as a key upstream activator of AKT [44]. Activated AKT reduces apoptosis by inhibiting the activity of the pro-apoptotic protein Bax, enhancing the expression of the anti-apoptotic protein Bcl-2, and suppressing the activity of caspase-3 [45,46,47]. In this study, phosphatidylinositol-3-kinase/protein kinase B (re)activation is nominal, although activity has been quantified via band intensity analysis. Our in vivo and in vitro results indicate that CTX-induced myelosuppression in mouse bone marrow cells and bone marrow mesenchymal stem cells is associated with decreased levels of p-PI3K/PI3K, p-AKT/AKT, and the anti-apoptotic protein Bcl-2, while the levels of pro-apoptotic proteins Bax and caspase-3 are increased. With increasing doses of VAPs, Bcl-2 levels were restored, and the expression levels of Bax and cleaved caspase-3 were reduced, whereas LY294002 reversed the therapeutic effects. In addition, peripheral blood cells in model mice were significantly reduced and BMNC counts were below normal values, while VAPs led to varying degrees of recovery in peripheral blood cells and increased the number of bone marrow nucleated cells. These findings suggest that inhibition of bone marrow cell apoptosis may be one of the important mechanisms through which VAPs treat myelosuppression and improve bone marrow hematopoiesis.

**Figure 15:**
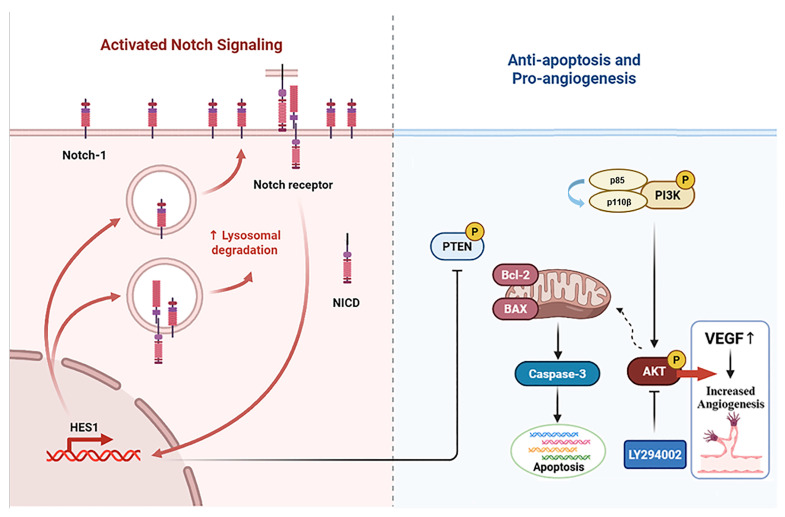
Schematic diagram of the mechanism through which VAPs alleviate bone marrow suppression through the Notch1/PI3K/Akt signaling pathway.

It is noteworthy that Notch1 plays an important role in the regulation of PI3K-AKT signal transduction [48]. The Notch pathway is a highly conserved intercellular signaling pathway involved in biological processes like the hematopoietic system, the nervous system, the muscular system, and the blood vessel system [49,50,51]. The Notch signaling pathway interacts with the PI3K/AKT signaling pathway through various mechanisms, including downstream target genes and cytokines, and it plays a significant role in multimorbidity [52,53]. Our experimental results showed that the expression levels of Notch1 and Hes1 proteins were decreased in the femur tissue of myelosuppression mice and VAPs upregulated the expression of Notch1 and Hes1 proteins in a dose-dependent manner, with the high dose of velvet antler polypeptides exhibiting the most significant effect. However, DAPT not only blocks the activation effect of VAPs on Notch1/Hes1 but also inhibits its reparative effect on the PI3K/AKT pathway. This confirms that Notch1, as an upstream regulatory factor of the PI3K/AKT pathway, mediates the bone marrow protective effect of VAPs (Figure 15).

## 5. Conclusions

In summary, our study demonstrates that VAPs promote the repair of bone marrow hematopoietic function by inhibiting bone marrow cell apoptosis, regulating hematopoietic factors within the bone marrow microenvironment, increasing their levels, enhancing angiogenesis, and restoring damage to the hematopoietic niche. Furthermore, it reveals that VAPs exert their hematopoietic-promoting effects by modulating the Notch1 and PI3K/AKT signaling pathways. This study provides an important scientific basis and identifies novel potential therapeutic targets for the application of VAPs in treating myelosuppression. It considerably enriches the modern understanding of the pharmacological activities of velvet antler. These findings not only deepen our theoretical knowledge but also lay a crucial foundation for the subsequent development of velvet antler as a hematopoiesis-promoting drug.

## Figures and Tables

**Table 1:** Amino acid composition of VAPs.

Amino Acids	RT (min)	Conc (mg/g)
Asp	8.387	57.187 ± 0.011
Thr	10.251	27.231 ± 0.050
Ser	11.140	32.090 ± 0.079
Glu	13.276	92.835 ± 0.007
Pro	14.672	52.799 ± 0.113
Gly	19.257	85.121 ± 0.091
Ala	20.38	49.977 ± 0.103
Cys	22.141	8.907 ± 0.023
Val	22.664	28.016 ± 0.150
Met	24.507	9.091 ± 0.068
Ile	25.828	16.793 ± 0.142
Leu	26.813	41.994 ± 0.019
Tyr	29.687	19.473 ± 0.073
Phe	30.507	26.993 ± 0.081
His	34.697	15.178 ± 0.138
Lys	37.337	40.975 ± 0.064
Arg	44.580	49.130 ± 0.0355

## Data Availability

The original contributions presented in this study are included in the article. Further inquiries can be directed to the corresponding author.
